# Cardiovascular Involvement in Erdheim–Chester Disease: A Case Report and Review of the Literature

**DOI:** 10.1097/MD.0000000000001365

**Published:** 2015-10-30

**Authors:** Maria Anna Nicolazzi, Annamaria Carnicelli, Mariella Fuorlo, Angela Maria Rita Favuzzi, Raffaele Landolfi

**Affiliations:** From the Institute of Internal Medicine, Catholic University of Rome, Rome, Italy.

## Abstract

Erdheim–Chester disease (ECD) is a rare, multiorgan, non-Langerhans cell histiocytosis of uncertain origin, characterized by systemic xanthogranulomatous infiltration from CD68+CD1a- histiocytes. Skeletal involvement is present in up to 96% of cases with bilateral osteosclerosis of meta-diaphysis of long bones. Furthermore, in more than 50% of cases there is 1 extraskeletal manifestation. In this case report, we describe an interesting case of ECD with an extensive pan-cardiac and vascular involvement, in addition to skeletal, retro-orbital, and retroperitoneum one.

A 44-year-old woman with a long history of exophthalmos referred to our hospital for elective surgical orbital decompression. At preoperative examinations a large pericardial effusion was discovered. Echocardiography, computed tomography (CT), and magnetic resonance imaging (MRI) described an inhomogeneous mass involving pericardium and the right heart, abdominal aorta and its main branches and the retroperitoneum, suggestive for a systemic inflammatory disorder. Histological examination on a biopsy sample confirmed the diagnosis of ECD. Radiology showed the pathognomonic long-bone involvement. Surgical orbital decompression was performed and medical therapy with interferon-α (INF-α) was started.

Among extraskeletal manifestations of ECD, cardiovascular involvement is often asymptomatic and thus under-diagnosed but linked to poor prognosis. This is why clinician should always look for it when a new case of ECD is diagnosed.

## INTRODUCTION

Erdheim–Chester Disease (ECD) is a rare, multiorgan, non-Langerhans cell histiocytosis. Approximately 550 cases have been described worldwide in the literature, with an increased diagnosis in the last 10 years. The age of incidence is between the 4th and the 7th decade of life, even if cases have been diagnosed between 7- and 84-year-old patients, with a slight male prevalence.^1^

Etiology is still unknown even if evidence of BRAFV600E mutation in ECD histiocytes, observed in more than half of cases, suggests a clonal nature of the disease.^2^ Furthermore, an association with TH1 lymphocytes activation and proinflammatory interleukins production has been observed. Elevated serum levels of interferon-α (IFN-α), interleukin (IL)-12, monocyte chemotactic protein-1 have been observed in ECD patients. These proinflammatory interleukins are probably responsible of local recruitment and activation of histiocytes.^3^

ECD indeed is characterized by tissue infiltration by foamy non-Langerhans histiocytes surrounded by fibrosis, which leads to a systemic xanthogranulomatous infiltration. At immunohistochemical analysis the involved histiocytes are typically CD68+ but CD1a- and S100- with the lack of Birbeck granules, unlike the Langerhans Cell Histiocytosis (LCH) where histiocytes are typically CD68+ and CD1a+.^4^

Skeletal involvement is present almost in all patients with symmetrical and bilateral osteosclerosis of metaphysis and diaphysis of long bones. In more than half of cases there is 1 extraskeletal manifestation. Cardiac involvement is often underdiagnosed and linked to poor prognosis.^5^

In this case report, we describe an interesting case of ECD with pan-cardiac and vascular involvement, in addition to skeletal, retro-orbital, and retroperitoneum one.

### Case Report

**FIGURE 1:**
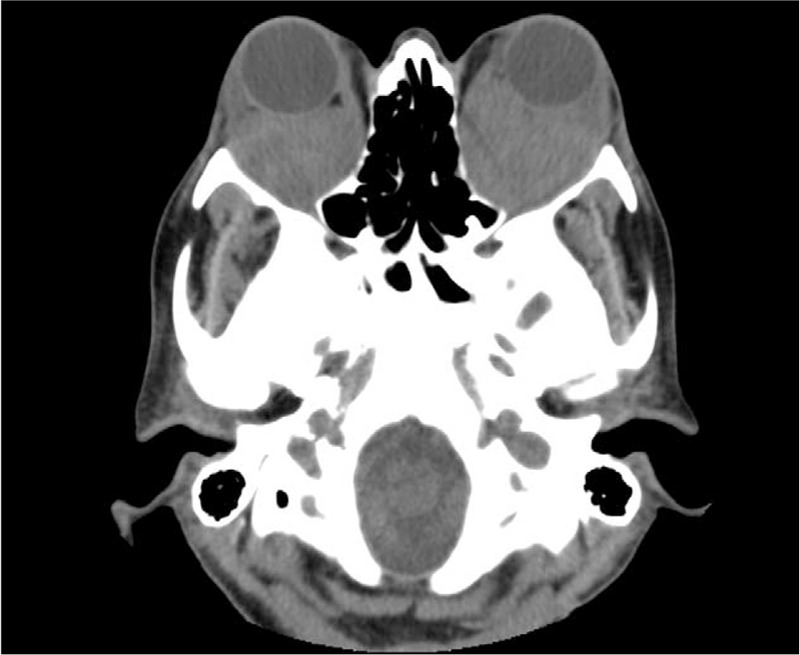
Axial cerebral computed tomography scan image of bilateral retro-orbital tissue causing exophthalmos.

A 44-year-old woman presented to our attention for a 4-year history of worsening bilateral exophthalmos. A biopsy of retro-orbital tissue performed 2 years earlier showed an inflammatory infiltration with an important fibrotic component; thus she was treated for a long period with corticosteroid and mycophenolate mofetil unsuccessfully. Therefore, she was referred to our institution for elective surgical intervention of orbital decompression (Fig. 1).

Except for hypothyroidism well treated with replacement therapy, she had no other medical chronic illness. She complained weakness but denied fever or any other significant symptom. Her past medical history was negative and she had no family history of cardiovascular, neoplastic, or autoimmune diseases.

At preoperative inspection vital signs were normal with blood pressure (BP) 110/60 mm Hg, heart rate (HR) 97 beats per minute, oxygen saturation (SO_2_) 97%. Physical examination was also normal, except or exophthalmos and mild peripheral edema. Blood tests showed slight normochromic–normocytic anemia, elevated erythrocyte sedimentation rate, and high serum creatinine level. A chest radiograph revealed a large cardiac silhouette and bilateral pleural effusion, while the electrocardiogram (EKG) showed sinus rhythm with low voltage.

**FIGURE 2:**
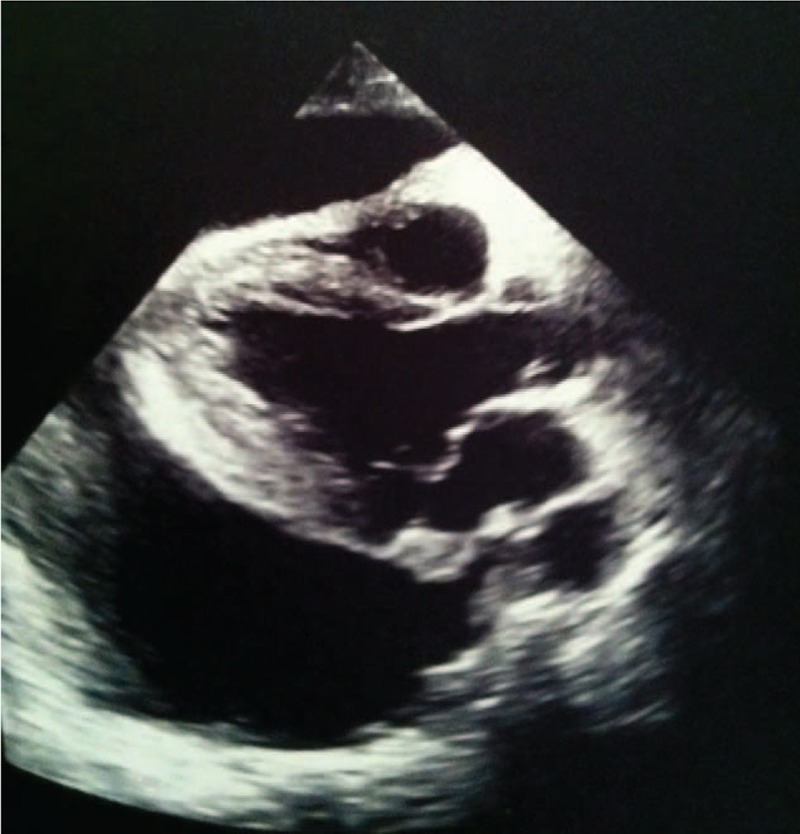
Transthoracic echocardiography: parasternal long axis view showing large pericardial effusion.

A transthoracic echocardiography revealed a large pericardial effusion with abnormal right heart filling and suggested the presence of a round hyperechoic and inhomogeneous mass attached to right atrial wall, extending to involve interatrial septum, atrioventricular groove, and right ventricle. Left ventricular ejection fraction (EF) was slightly reduced (Fig. 2). The patient was subjected to diagnostic and therapeutic pericardiocentesis with drainage of about 1200 mL of serous fluid. Microbiological and cytological examinations on pericardial fluid resulted negative.

**FIGURE 3:**
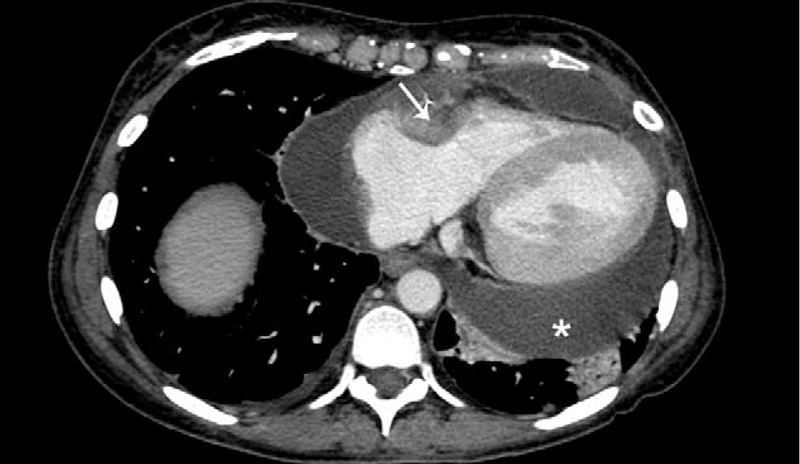
Axial contrast-enhanced computed tomography scan image of the chest showing pericardial effusion (^∗^) and soft tissue involving right atrioventricular groove, extending to right ventricular free wall and right atria (arrow).

A contrast-enhanced chest computed tomography (CT) scan postpericardiocentesis showed residual pericardial effusion, smaller pleural effusion and evidenced a contrast enhancing solid tissue involving right atrioventricular groove, on epicardial side, surrounding right coronary artery without obstruction and extending to right atrial wall until superior vena cava entry (Fig. 3).

Moreover, a cardiac magnetic resonance imaging (MRI) confirmed the presence of the tissue (39 × 32 mm on axial plane) with focus on the epicardial side of atrioventricular groove extending caudally and cranially to contact aortic root and interesting right atrial wall and right ventricular free wall. Laterally the mass extended until visceral pericardium.

Considering both the retro-orbital and the cardiac involvement, a systemic disease was hypothesized. Particularly, the typical cardiac involvement suggested a chronic inflammatory disorder.

**FIGURE 4:**
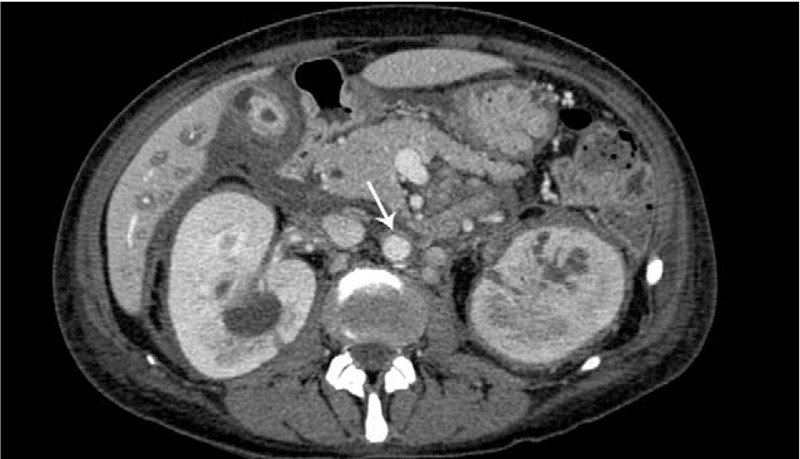
Axial contrast-enhanced abdominal computed tomography scan image in a 44-year-old woman, showing bilateral and symmetrical infiltration of the adrenals and renal sinuses with right hydronephrosis. Note also soft tissue rounding portal vein until liver hilus and intrahepatic branches. Periaortic concentric soft tissue, also known as “coated aorta,” is visible (arrow).

The patient was therefore subjected to abdomen and pelvic contrast-enhanced CT scan with evidence of an extensive retroperitoneum tissue rounding abdominal aorta until iliac bifurcation, renal, celiac and mesenteric arteries, inferior cava vein and portal vein. In addition, the tissue involved liver hilus, both kidneys and adrenals with severe right hydronephrosis that needed percutaneous nephrostomy (Fig. 4).

During the hospitalization a surgical intervention of orbital decompression was performed and a retro-orbital tissue sample was collected. A new histological examination evidenced the presence of fibrosis and an inflammatory infiltration of foamy histiocytes. Particularly, at immunohistochemical analysis they were CD68 positive and CD1a and S100 negative, as is described in ECD. BRAFV600E mutation was absent.

**FIGURE 5:**
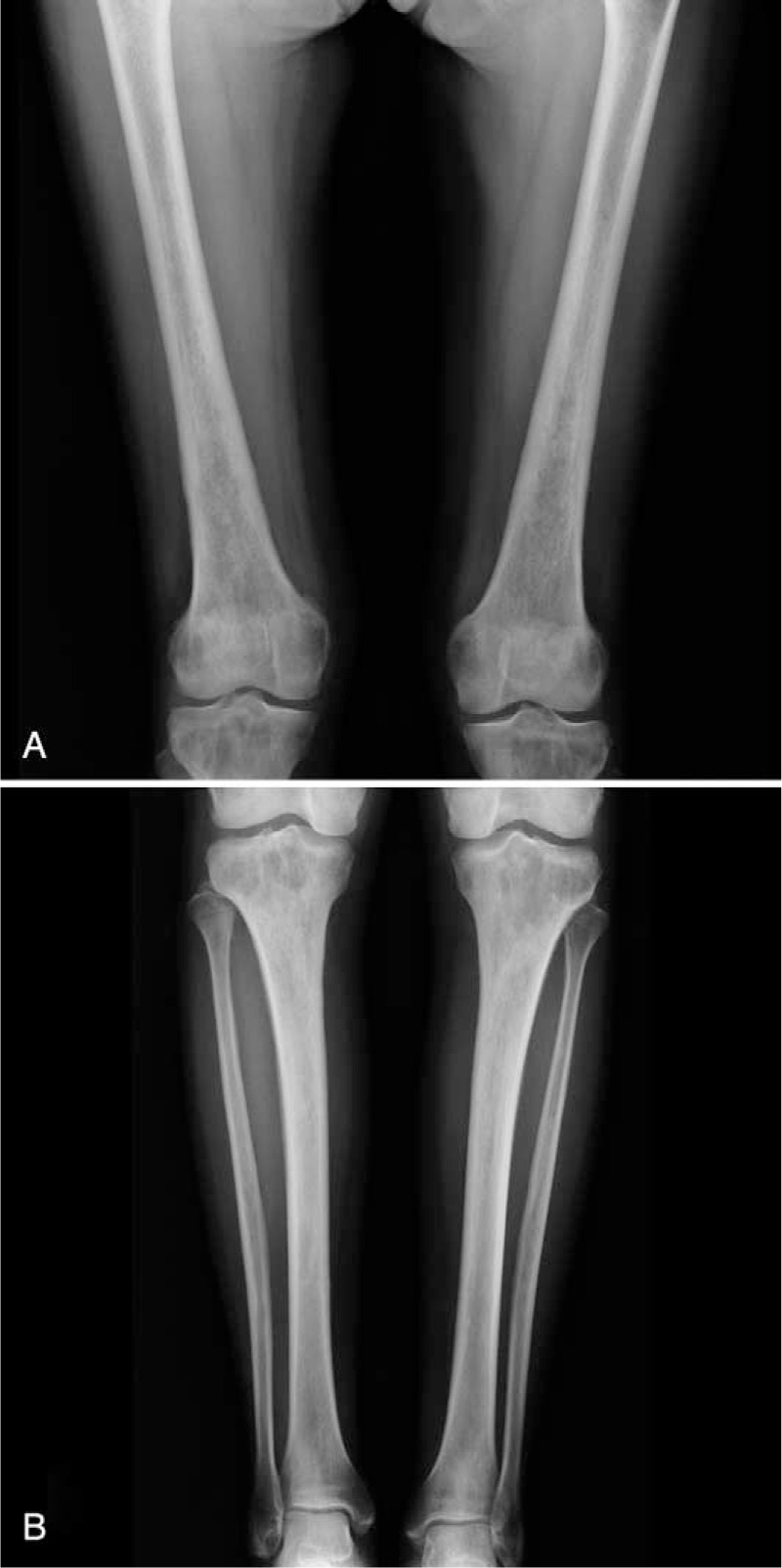
Femur (A), tibia and fibulas (B) radiography showing bilateral and symmetrical osteosclerosis of metaphysis and diaphysis.

To complete the diagnostic process, a long bone radiography and scintigraphy showed a pathognomonic bilateral symmetrical osteosclerosis of dia-metaphysis of long bones of upper and lower limb (Fig. 5) and of mandible. Cranial contrast-enhanced MRI excluded pituitary or cerebral involvement and confirmed the retro-orbital tissue.

A treatment with interferon-α (INF-α) in association with corticosteroids and colchicines was started. At follow-up visits a substantial stability of manifestations and reduction in disease progression was observed.

## DISCUSSION

ECD is a rare disease with very heterogeneous manifestations, from an asymptomatic and limited organ involvement to a massive and life-threatening disease. Because of its systemic presentation, ECD can be associated with general and unspecific symptoms such as weakness, fever, weight loss, sweats in addition to multiorgan affection. Skeletal involvement is present in up to 96% of the patients with symmetrical and bilateral osteosclerosis of metaphysis and diaphysis of long bones, sparing epiphysis, and axial skeleton. Despite these pathognomonic radiological features, only half of the patients complain about bone pain. Moreover, more than 50% of patients have at least 1 extraskeletal manifestation, such as exophthalmos, xanthelasma, interstitial lung disease, retroperitoneal fibrosis, renal failure, diabetes insipidus, central nervous system, or cardiovascular involvement.^6–8^

Radiological features are often typical but histological examination is required for definitive diagnosis with evidence of CD68+ but CD1a- foamy histiocytes with fibrosis. CD68 is a specific histiocyte marker and CD1a is a marker for Langerhans cells; this finding distinguishes ECD from LCH.^9^

In our case report, we describe a case of multiorgan ECD with an extraordinary pan-cardiac and vascular involvement, involving the pericardium with large pericardial effusion, myocardium, right atria and ventricle, abdominal aorta and its branches, superior and inferior vena cava, and portal vein.

Interestingly, our patient presented a long history of a misdiagnosed exophthalmos associated with retro-orbital pseudotumor, in the absence of other relevant constitutional or cardiovascular symptoms. Further radiological examinations evidenced the pathognomonic skeletal involvement, in the absence of bone pain, and an extensive retroperitoneum tissue.

Cardiovascular involvement in ECD is common but not always clinically evident, often asymptomatic and so underdiagnosed. As described by Haroche et al, among all patients with cardiovascular involvement, cardiac one is present in about 75% of cases and is strictly linked with poor prognosis. Death is due to cardiovascular involvement in more than one-third of cases and this is why physician should always look for it when a new case of ECD is diagnosed.^7,10^

The most common cardiovascular manifestation in ECD is the presence of soft tissue rounding thoracic and abdominal aorta and its branches, also known as “coated aorta,” as we have described in our patient.^11^ With regard to heart involvement, the most frequent feature is pericardial infiltration that may lead to cardiac tamponade.^12,13^ Second, myocardial localized or diffuse infiltration is often observed with frequent pseudotumoral infiltration of right atrium and involvement of the auricoloventricular sulcus.^14,15^ Myocardial infiltration, in association with pericardial one, were both present in our patient. Moreover, coronary disease with myocardial infarction, valvular dysfunction, and heart failure have been described in the literature.^16–19^

To date, several therapeutic approaches to ECD have been proposed, but only few prospective therapeutic studies have been done. Actually, the most effective therapeutic strategy is considered INF-α and Pegylated IFN-a (PEG-IFN-a).^20^ Although the optimal dosage has not been established yet, generally INF-α is administered at dosages ranging from 3 to 9 million units 3 times per week while PEG-INF-α is administered at dosages ranging from 135 to 200 μg per week. Also the optimal duration of treatment is not clear but long-term therapy (up to 3 years) has been associated with greater efficacy in terms of stabilization and improvement of the disease. Moreover, treatment with INF-α has been identified as a predictor of survival in ECD patients.^21,22^

Recent advancements in the understanding of the molecular biology of ECD and on the proinflammatory role of interleukins encouraged the use of cytokine antagonist therapies, which, however, resulted effective in a small number of reported patients.^23^

Corticosteroids may be useful, in combined therapy, to reduce edema but they are not effective in reducing disease progression. Radiotherapy and chemotherapy use has been reported but with no important results, while surgical approach is limited to resectable masses.^1,6^ Today, a new treatment with Vemurafenib (an inhibitor of BRAF harboring the V600E mutation) has been proposed with interesting results, but new prospective trials are needed.^24^
